# Integrative Wellness Approaches to Mitigate Perceived Stress, Increase Vitality, and Build Community during COVID-19: A Pilot Study

**DOI:** 10.3390/ijerph192416463

**Published:** 2022-12-08

**Authors:** Amber L. Vermeesch, Layla Garrigues, Chloé Littzen-Brown

**Affiliations:** 1 Department of Family and Community Health, School of Nursing, University of North Carolina Greensboro, Greensboro, NC 27402, USA; 2 University of Portland School of Nursing & Health Innovations, Portland, OR 97203, USA

**Keywords:** subjective vitality, community building, perceived stress, integrative health, wellness interventions, burnout, compassion fatigue, nurse educators, COVID-19

## Abstract

Introduction: In January 2020, a small, private school of nursing in a university in the pacific northwest, established the Initiative for Vital Practice (I4VP). The I4VP’s primary goal was to create a sustainable pathway for increasing vital practice through increasing resiliency and self-care practices. Objectives. The ensuing pathway’s objectives were to, (1) take previously identified factors related to perceived stress related to workloads, impacts on professional quality of life and psychosocial exposures during the COVID-19 pandemic; and (2) develop and pilot test a wellness intervention (i.e., wellness pods) for faculty and staff to build community and find new ways to enhance well-being through peer support. Methods: Five focused Wellness Pods were developed on Microsoft Teams platform using the individual channels: (1) stress and mind–body exploration pod; (2) mindfulness in healthcare pod; (3) healing relationship pod; (4) environmental pod; and (5) physical activity pod. Faculty and staff self-selected into a Wellness Pod that interested them. The Wellness Pods met weekly in person over a period of two months. Quantitative and qualitative data was collected via cross-sectional surveys including: four sociodemographic items, one item on current stress level, one write-in item on current stress management at work, two write-in items focused on the cognitive reasoning for participation, the 7-item subjective vitality scale focused individual difference, the 7-item subjective vitality scale focused on the state level, the 10-item perceived stress scale, and one item ranking which wellness pod the individual wanted to participate in. There was one trained facilitator for the overall Wellness Pods operations and communication. Results. The average score on the perceived stress scale was 22.3 (SD = 3.5), indicating moderate levels of perceived stress. The average score on the individual difference vitality score was 26.5 (SD = 7.6), whereas the state level vitality score was 21.4 (SD = 9.98), indicating moderate levels of subjective vitality. Two categories: stress management and wellness pods, were identified through content analysis. Conclusions: Through pilot testing, this project demonstrated feasibility for future wellness pods interventions for faculty and staff at schools of nursing. Future research is needed to evaluate the effectiveness of the wellness pods intervention.

## 1. Introduction

In January 2020, in response to signs of an increase in perceived stress among faculty, staff, and students, a small, private school of nursing in a university in the pacific northwest, established the Initiative for Vital Practice (I4VP). Although the I4VP was created prior to the COVID-19 pandemic’s commencement, we were seeing physical and mental manifestations of perceived stress rising exponentially. The I4VP’s primary goal was to create a sustainable pathway for increasing vital practice, defined as the engagement in systematic reflection and self-care activities, thereby increasing resiliency for university workers and students. The vital practitioner is defined as a caregiving professional who engages in systematic self-care activities designed to increase subjective vitality (e.g., time management, forest bathing) thereby mitigating perceived stress by increasing subjective vitality. Perceived stress and subjective vitality are important to address as they contribute to occupational longevity, job performance, psychosocial conditions, and physical manifestations that impact nurse connection with patients [1,2]. Thus far, the I4VP has utilized online workshops, art therapy, narrative writing, discussions, and professional quality of life to accomplish its mission. The purpose of this paper was to describe the development and pilot-testing of a wellness intervention, along the ensuing pathway, to reduce perceived stress, ultimately increasing subjective vitality. The wellness intervention, titled wellness pods, was conducted between June and December of 2021 during the COVID-19 pandemic. Prior to this intervention, there was not an established pathway for peer supported wellness for faculty and staff at this institution. In previous work by the I4VP, wellness pods were identified by the faculty and staff as a potentially sustainable and effective intervention, but pilot testing was needed, especially to determine faculty and staff buy in and readiness. This paper contributes to the literature by determining the feasibility of a novel, buddy-system approach, wellness intervention.

### Background

Compassion fatigue, or the cost of caring, is a stress injury for nurses and other caregiving professionals [3]. First defined by Joinson (1992) [4] as the loss of the ability to nurture, compassion fatigue is an important concept in occupational health research linked to both individual and organizational outcomes. Compassion fatigue, a component of perceived stress, is often conflated with burnout, a psychological phenomenon characterized by, (1) emotional exhaustion (feeling emotionally drained and lacking emotional resources), (2) depersonalization (having a negative and detached response to other people along with a lack of idealism), and (3) reduced personal accomplishment (a decline in feeling competent in job related performance) resulting from excessive stress at work, but they are inherently different [5,6]. According to Boyle (2011) [7], compassion fatigue is different from burnout as it acutely occurs when the “rescue-caretaking strategies are unsuccessful, leading to feelings of distress and guilt,” making it is of relational concern. Comparatively, burnout stems from conflict in the work-setting related to conflicts with managers or inadequate working conditions and is more chronic in nature [7]. Burnout is of concern to nursing occupational health as it lowers nurses’ quality of life, job performance, organization commitment while increasing intention to leave and turnover rates, ultimately negatively impact the quality of nursing care [8]. Perceived stress, which is defined as a person’s reaction to an environment that is perceived as a threat to their abilities and/or health [9], has the greatest influence on the burnout of nurses compared to any other factor [10,11]. Subjective vitality is an influential factor to combat compassion fatigue and perceived stress [2]. Perceived stress, which is the primary proxy for the impacts on burnout as described above, was the focus of this feasibility study for the vital practitioner and were highlighted in importance during the COVID-19 pandemic.

The literature on nursing faculty perceived stress and subjective vitality, as presented above, is substantially growing especially with the looming higher educational crises [12]. Nursing faculty and staff are regularly asked to do more with less resources in addition to the recent challenges of the COVID-19 pandemic. This approach to education is not sustainable moving forward. With national wide nursing shortages of both faculty and practitioners [13] the nursing discipline has been at a breaking point for over 30 years [14]. This precipice effectively sheared off and broke during the COVID-19 pandemic with the compounding of current crises in higher education [12,15]. The nursing profession cannot go back to ‘business as normal’ as it existed before the COVID-19 pandemic. It will be integral to establish new pathways in nursing education and practice that mitigate perceived stress and increase subjective vitality. As an example of this critical need, the American Association of Colleges of Nursing (AACN) released in February 2022 the New Essentials [16] that outline the importance of self-care, well-being, resilience, and leadership development training for baccalaureate nursing students and programs. To be better prepared to teach these new essentials, nursing faculty and with the support of nursing staff, need to be well versed in the importance and practices of to manage and enhance their being–whether that is through self-care or resilience training.

For this feasibility study, we intend for there to be positive impacts at both the individual and institutional levels. Further, by generating new knowledge on the mechanics of stress, I4VP is bridging theory to practice in academia and the caregiving field. Thus, this pilot study had two objectives: (1) take previously identified factors related to perceived stress related to workloads, impacts on professional quality of life and psychosocial exposures during the COVID-19 pandemic; and (2) develop and pilot test a wellness intervention (i.e., wellness pods) for faculty and staff to build community and find new ways to enhance well-being through peer support

## 2. Materials and Methods

### 2.1. Pilot Study Methods

Previously identified factors related to perceived stress included lack of collegiality and sense of community, low salaries, long working hours, lack of support and lack of social support, few opportunities for advancement and growth in their jobs, lack of control over work-place decisions, and not enough time to rest [17,18]. Guided by these previously identified factors, this study used a feasibility implementation with cross-sectional electronic surveys including both quantitative and qualitative responses. The rationale for this design was to determine feasibility of a peer supported electronic well-being intervention. In this study, Wellness Pods for the university school of nursing faculty and staff in 5 core areas were developed: (1) stress and mind–body exploration including mindfulness and stress reduction, (2) mindfulness in healthcare including how be better aligned with mindfulness techniques in healthcare, (3) healing relationship–beginning with self and self-compassion and including communication, (4) environment–tips and techniques to create healing environment as well as excursions into local nature areas, (5) physical activity–accountability to other pod members. The intervention for implementation was the development of Wellness Pods and inviting faculty and staff to join the pod of their interest. Wellness pods are a group (or pod) of individuals who are focused on a specific area of wellness; in our case, our wellness pods consisted of university faculty and staff. The 5 core integrative areas were chosen by the primary investigator and main facilitator after completing the University of Arizona Integrative Health and Lifestyle program (IHeLp) and the Integrative Nursing Faculty Fellowship (INFF) programs through the Andrew Weil Center for Integrative Medicine [19] to guide the wellness pods. The IHELP fellowship program determined these five areas as imperative to integrative health and lifestyle strategies for faculty and staff. 

### 2.2. Setting and Sample

The setting was at the University of Portland School of Nursing and Health Innovations, which is a private non-profit University in the pacific northwest of the United States. The dates of this wellness intervention were between June and October of 2021 during the COVID-19 pandemic. Using convenience sampling using fliers, emails, and word of mouth, targeting university school of nursing staff, tenure-track faculty, non-tenure track faculty, and leadership were invited to participate to determine how and what the organization can do as a system to improve the workplace environment and create the conditions for increased vitality. Inclusion criteria for participation included being a full-time employee as either a faculty or staff member. Exclusion criteria included not being a full-time employee as either a faculty or staff member.

### 2.3. Procedures

Through the I4VP, we created and implemented a wellness intervention, named wellness pods in the five core integrative areas aforementioned. As previously described, wellness pods are a group (or pod) of individuals who are focused on a specific area of wellness; in our case, our wellness pods consisted of university faculty and staff. Faculty and staff self-selected into a wellness pod that interested them. Wellness pods are designed to be self-sustainable, self-directed, and members of the wellness pods have accountability to other pod members. The wellness pods met in person weekly over a period of eight weeks. Microsoft Teams [20] pages were used as the main communication channels for the wellness pods. Within these Teams pages, there were individual channels for each Wellness Pod, and each Teams page housed specific information related to that pod. 

### 2.4. Data Collection

Data was collected over eight weeks, during fall semester between September and October 2021. Thirty-three items were delivered to the participants online via Qualtrics XM [21]. Responses were anonymous and the following questions were embedded in Qualtrics and used to aid participants in creating a unique code to protect participant identity.

Only individuals who consented to participating in the IRB approved program could access and complete the survey. The survey included four sociodemographic items, one item on current stress level, one write-in item on current stress management at work, two write-in items focused on the cognitive reasoning for participation, the 7-item subjective vitality scale focused individual difference [22], the 7-item subjective vitality scale focused on the state level, the 10-item perceived stress scale [23], and one item ranking which wellness pod the individual wanted to participate in. 

### 2.5. Subjective Vitality Scale (SVS)

The 7-item subjective vitality scale was developed by Ryan and Frederick [22] to measure subjective vitality based upon self-determination theory. This scale was appropriate for this study as it captures subjective vitality which is a vital component of well-being and should be addressed with wellness interventions. Questions are rated on a seven-point scale from “not true at all” to “very true.” Original psychometric testing of the scale demonstrated strong internal consistency reliability with a Cronbach’s alpha at 0.84. The scale was also found to have positive correlations with other related concepts of well-being such as self-actualization, self-esteem, and satisfaction with life [24]. Comparably, negative correlations have been found between subjective vitality and oppositional concepts of depression, negative affect, anxiety, as well as measures of psychopathology [24]. The scale is scored by averaging the individual items, and it is recommended to omit item #2 for analysis [25]. Total scores can range from 6–36. Higher scores indicate higher levels of subjective vitality, whereas lower scores indicate lower levels of subjective vitality.

### 2.6. Perceived Stress Scale (PSS)

The perceived stress scale (PSS-10) originally developed by Cohen et al., [23], is a 10-item questionnaire widely used to assess the stress levels in youth and adults 12 years and older. This scale was appropriate for this research to determine, using a validated scale, perceived stress levels of faculty and staff to therefore eventually determine effectiveness of wellness interventions. Questions are rated on a 4-point scale, from “never” to “very often” [23]. Original psychometric testing of the scale demonstrated good internal consistency reliability with a Cronbach’s alpha of 0.78, test–retest reliability, construct validity, convergent validity, and concurrent validity [26]. Total score of the PSS is determined by reversing all positively stated items (items 4, 5, 7, and 8), then summing across all items. Total scores can range from 0–40. Scores that range from 0–13 are considered low levels of perceived stress, 14–26 are moderate levels of perceived stress, and 27 to 40 are high levels of perceived stress. 

### 2.7. Data Analysis

Project outcomes were measured through data analysis of the survey to determine feasibility of the wellness pods intervention. The de-identified data was analyzed in Microsoft Excel [20] for basic descriptive statistics and measures of central tendency. Content analysis per the method of Elo and Kyngäs [27] was followed for the three qualitative write-in questions. Two separate investigators reviewed and analyzed the qualitative data. Trustworthiness per the standards of Lincoln and Guba [28] were followed.

## 3. Results

Twenty-two individuals consented to participate in the survey prior to implementation of the wellness pods. There were a total of 77 faculty and staff that met inclusion criteria resulting in a response rate of 29%. Of the 22 who gave consent, 16 completed the survey, or approximately 73% completion and adherence rate. Three participants consented to the survey but did not complete any survey items. Three participants only completed the first three demographic items after consenting to the survey. Data were analyzed for missingness which did not appear systematically related to any items in the survey. All available responses were included in the data analysis. Feasibility of this novel, buddy-system approach, wellness intervention was determined thus adding to the literature on sustainable wellness interventions in building community and ways to support well-being. 

### 3.1. Sociodemographics

Participants primarily identified as female (n = 17, 89%), with an average age of 50–55 years (n = 5, 26%). For academic appointments, 53% (n = 10) of the participants were instructors, 37% (n = 7) were tenure-track, 5% (n = 1) were senior lecturers, and 5% (n = 1) were staff members. The average years of experience was 10+ years (n = 4, 21.5%). Table 1 presents the sociodemographic summary.

### 3.2. Quantitative Results

Pre-wellness pods, on a scale of 1 to 5 (1 being no stress and 5 being very stressed), on average, 37% of participants rated their stress at 3.31 (SD = 1.04) indicating a midline amount of stress. The average score on the perceived stress scale was 22.3 (SD = 3.5), indicating moderate levels of perceived stress. The average score on the individual difference vitality score was 26.5 (SD = 7.6), whereas the state level vitality score was 21.4 (SD = 9.98), indicating moderate levels of subjective vitality. 

### 3.3. Qualitative Results

**Figure 1:**
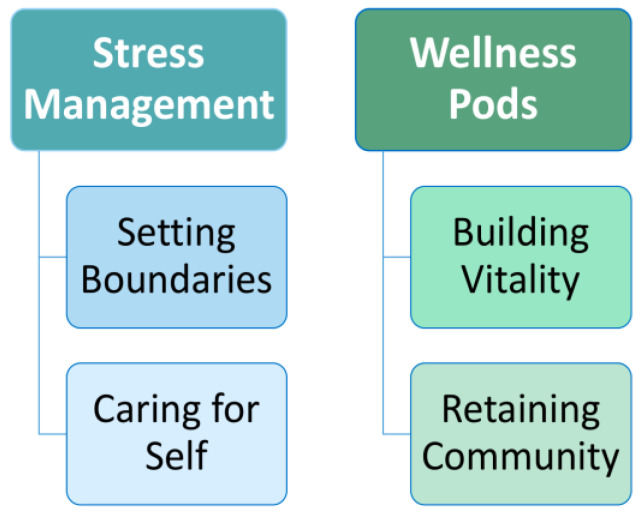
Qualitative Analysis: Categories and Subcategories.

Qualitative content analysis was completed on pre-wellness pods surveys [27] of the three write-in questions resulted in the identification of two categories: stress management and wellness pods. Stress management, which is defined as strategies for dealing with difficulties and challenges in life and work, included the subcategories of setting boundaries and caring for self. Wellness pods, which were defined as groups of like-minded people working and collaborating for revitalization and well-being, included the subcategories of building vitality and retaining community (Figure 1). 

### 3.4. Stress Management

For the category of “stress management,” the subcategory “setting boundaries” was defined as learning and practicing a life for protecting personal values and preventing them from being compromised. Setting boundaries was important for stress management and involved not working on weekends and decreasing work screen time at home. Participants highlighted prioritizing time within their boundary setting, focusing on family, exercise, and friends. The subcategory “caring for self” was defined as the ability and practice of promoting well-being for the individual, included physical exercise (e.g., running, biking, walking, dance), being outdoors (e.g., pruning plants, gardening, and mushroom hunting), and nutrition and adequate sleep, mindfulness, meditation, journaling, deep breathing/exercises, laughter, art, music, reading, interacting with animals, and watching movies for stress reduction.

### 3.5. Wellness Pods

For the category of “wellness pods,” the subcategory of “building vitality” defined as the ability to grow, develop, and energize with enthusiasm and vigor, was important to prevent burnout, decrease stress, improve well-being, and work/life balance. This is important as participants described wanting to learn ways to manage and address stress with a focus on stress reduction and improvement in quality of life. Participants described wanting to, “increase tools for stress management” that is part of self-care and to not take out stress on their families. One participant described how they have had previous experience with burnout, and that they are now motivated to build skills and to not be burned out. There was curiosity in wanting to learn breathing and relaxation techniques for self-care and to teach students.

For the category of “wellness pods”, the subcategory of “building community”, participants describe wanting to network with colleagues, build connections, and understand their workplace better. This is a new type of engagement in the community that is an innovative work-place balance in decreasing stress, fostering comfort with vulnerability, and building a sense of safety in the workplace community. This community building involves self-agency and knowledge building. Participants want the workplace to be stress-relieving, they want to perform better in relationships and at work to develop a sense of community working in groups or wellness pods, gaining a sense of support from colleagues at work, as well as contributing to the community and making a difference. There were also descriptions of working through personal hesitation about vulnerability in the workplace which can be challenging. One participant stated, “I feel like I am barely treading water with my wellness; I need community.” Participants discussed the goal of improving working conditions and faculty morale. There is importance in contributing to future solutions to decrease stress, reduce faculty burnout, and retain faculty. Participants describe how engaging in wellness pods would help maintain professional connections, improve working conditions, and faculty morale.

## 4. Discussion

The primary findings from this pilot study demonstrate that the wellness pods intervention is a feasible approach to enhance the perceived stress and subjective vitality of nursing faculty and staff, but more research is needed. Due to the constraints of the COVID-19 pandemic, a more robust pilot study was not possible. Future research is recommended to repeat this intervention in other settings with repeated measures to determine change after intervention implementation. At this time, this study lays the foundation and baseline for future work on wellness pod interventions within the population of nursing faculty and staff. 

The objectives of this study were to: (1) take previously identified factors related to perceived stress related to workloads, impacts on professional quality of life and psychosocial exposures during the COVID-19 pandemic; and (2) develop and pilot test a wellness intervention (i.e., wellness pods) for faculty and staff to build community and find new ways to enhance well-being through peer support. According to the Future of Nursing 2020–2030 goals [22], nursing faculty and staff need support in their health and well-being. The categories of “stress management” and “wellness pods” provide qualitative support for approaches to reduce perceived stress, build community, and enhance well-being in faculty and staff. The two categories of “stress management” and “wellness pods” provide evidence of the need for additional tools and trainings in the pursuit of health and wellness. This is why innovative wellness interventions, such as the wellness pods, are needed and why the Future of Nursing 2020–2030 has made health and well-being of nursing faculty one of their goals. 

### 4.1. Factors Related to Perceived Stress

In relation to objective one, previously identified stressors in the workplace for faculty and staff include lack of collegiality and sense of community, low salaries, long working hours, lack of support and lack of social support, few opportunities for advancement and growth in their jobs, lack of control over work-place decisions, and not enough time to rest [17,18]. In this study, we found similar results with faculty and staff wanting to network with colleagues, build connections, and understand their workplace better to promote their work-related well-being. Moreover, nursing faculty have been found to experience stress related to heavy and excessive workload of both courses taught and number of students, and increased pressure to publish and obtain external research grants while balancing teaching responsibilities [17,18,29].These stressors result in faculty and staff experiencing moral distress, burnout, compassion fatigue, insomnia, difficulty concentrating and focusing at work, decreased mental capacity for completing work tasks, issues around time management, feeling emotionally and physically drained, and increased absenteeism [30,31]. In our study, we found that faculty needed protective mechanisms to promote and enhance their work-related well-being, such as through setting boundaries and caring for themselves. This finding, while novel, demonstrates that faculty are having to integrate personal changes to manage the work environment indicating systemic changes may be needed. 

### 4.2. Previous Wellness Interventions to Build Community and Enhance Well-Being

In 2021, Melnyk et al. [32] substantiated the need for universities to assist faculty, staff, and students in seeking professional help through a variety of programs and the need to prioritize cultures of wellness. Previous wellness interventions evaluated a variety of different types of interventions for decreasing stress, increasing mindfulness and resilience, and improving mood, anxiety, sleep, and fatigue focusing on healthcare providers, not nursing faculty. Preceding interventions focus on supporting exercise, nutrition, cognitive behavioral therapy, mindfulness, and stress management for healthcare workers—not building community and enhancing well-being through peer support for nursing faculty and staff as stated in objective two of this study [33,34,35,36,37,38,39,40]. Woods-Giscombe [31] describes an innovated program to promote health promotion, quality of life, and wellness for School of Nursing faculty, staff, and students which includes elements of mindfulness, mind–body skills, hallway posters encouraging using the stairs for cardiovascular health, and wellness moments among others but does not utilize peer support to build community as a means of enhancing well-being.

### 4.3. Moving Forward with Theoretical Considerations

An overarching theory that addresses the categories and subcategories is the hope theory where there are three components associated with hope: (1) developing and having goals; (2) creating strategies to achieve goals; and (3) having the motivation to achieve goals [27]. Hope is defined as the “perceived capability to derive pathways to desired goals and motivate oneself via agency thinking to use those pathways” [41] (p. 249). Having hope involves remaining optimistic about the future and moderating negative thoughts and experiences. The wellness pods intervention has the potential to enhance participants’ subjective vitality and goal-oriented components of hope, a skill for imagining a positive future [41] by setting boundaries, caring for self, building vitality, and retaining community. Learning new strategies can help faculty and staff become more engaged at work and to better support their team. More specifically, developing a healthy work environment that is proactive and preventative in nature may ultimately lead to better quality of life for faculty, staff, and, in turn, a better learning experience for nursing students, improving subjective vitality. The outcomes of this pilot project address stress and mind–body exploration including mindfulness and stress reduction which are important components in developing a healing relationship that begins with the self, focusing on self-compassion and communication then expanding to wider spheres of interactions with others. Faculty and staff can set their personal goals addressing mindfulness, physical activity, and healing relationships which can act as a foundation in supporting students. 

### 4.4. Limitations

Limitations of this study include a small sample size of participants in an academic setting impacting generalizability. Future research is needed to determine the significance of the wellness pods intervention with a larger sample size in different settings. Self-reporting bias is a concern for participants due to the fact the primary investigator was a colleague at the university. Even though participants anonymously participated in the survey, the primary investigator knew which individuals were in each wellness pods and being a small sample size could have impacted individual’s authentic participation. Lastly, the COVID-19 pandemic was also a limitation as it proved difficult for participants to complete follow-up measures due to illness and scheduling. 

## 5. Conclusions

Based upon our objectives, we guided this study on previously identified factors related to perceived stress, workloads, impacts of professional quality of life, and psychosocial exposure during the COVID-19 pandemic. Findings indicate that while faculty and staff were experiencing moderate levels of perceived stress, they were desiring a means to build community and support their well-being. This brings us to our final objective, where we developed and piloted tested the wellness pods intervention to help faculty build community and enhance their well-being through peer support. Although we were unable to collect post-intervention data, our pilot demonstrated beginning feasibility for future wellness pods interventions for faculty and staff at schools of nursing. Future research is needed to evaluate the effectiveness of the wellness pods intervention.

To conclude, the I4VP, with continued efforts will greatly enhance, facilitate, and the growing work and concern around caregiver burnout, which includes nursing faculty and staff. This need is highlighted by the longitudinal COVID-19 pandemic impacts, and the increasing toll it is taking on faculty and staff. To create a sustainable pathway for increasing vital practice, integrative resources that university faculty, staff, and students can add to their toolbox include vital suggestions in the Chapter 18 *Rewilding Wellness: An Integrated Perspective*, which address environment and community aspects, health aspects such as nutrition and substance use, relationship aspects, wellness examinations, stress, functional medicine, and integrative health care [42]. In a world with a successful I4VP, health care workers, faculty, staff, students, and the systems in which they operate can add “vital practice” to their list of essential Personal Protective Equipment. 

## Figures and Tables

**Table 1:** Participant sociodemographics.

Category		Number of Participants N (%)
Gender Identity	Male	2 (11%)
	Female	17 (89%)
	Non-Binary	0 (0%)
	Total	19 (100%)
Age (years)	20–25	0 (0%)
	25–30	0 (0%)
	30–35	2 (11)
	35–40	4 (21%)
	40–45	3 (16%)
	45–50	3 (16%)
	50–55	5 (26%)
	55–60	1 (5%)
	60–65	1 (5%)
	65+	0 (0)
	Total	19 (100%)
Academic Appointment	Instructor	10 (53%)
	Tenure Track	7 (37%)
	Senior Lecturer	1 (5%)
	Staff	1 (5%)
	Other	0 (0%)
	Total	19 (100%)
Years of Experience	Less than One Year	3 (15.7%)
	1–2 Years	3 (15.7%)
	3–5 Years	3 (15.7%)
	6–9 Years	3 (15.7%)
	10 + Years	4 (21.5%)
	Missing	3 (15.7%)
	Total	19 (100%)

## Data Availability

Not applicable.
